# Structural and Functional Connectivity Between the Amygdala and Orbital Frontal Cortex in Burning Mouth Syndrome: An fMRI Study

**DOI:** 10.3389/fpsyg.2019.01700

**Published:** 2019-07-25

**Authors:** Ying Tan, Xunhua Wu, Jing Chen, Lingyu Kong, Zhaoxin Qian

**Affiliations:** ^1^Health Management Center, Xiangya Hospital, Central South University, Changsha, China; ^2^Department of Radiology, Central Xiangya Hospital, South University, Changsha, China; ^3^Center of Stomatology, Xiangya Hospital, Central South University, Changsha, China; ^4^Department of Emergency, Xiangya Hospital, Central South University, Changsha, China

**Keywords:** burning mouth syndrome, brain, functional connectivity, amygdala, ventromedial prefrontal cortex

## Abstract

Featuring a burning sensation in the tongue or other oral sites in the absence of observable lesions or laboratory findings, burning mouth syndrome (BMS) is a chronic intraoral pain disorder, which is one of the most common medically unexplained oral symptoms/syndromes. Previous studies have suggested that brain changes are involved in BMS; however, the small number of participants in these studies limited the conclusions that could be drawn. The present study aimed to further elucidate the brain anatomical and functional changes in BMS with a relatively large sample. Fifty-three patients (26 BMS patients and 27 gender- and age-matched controls) were recruited. Demographic information was collected *via* interviews. Visual analogue scale (VAS), anxiety, and depression scale were administered. Participants underwent an MRI scan (including one high-resolution structural scan, one diffusion tensor image, and one session of resting state scan) on the same day. The results showed that BMS patients had higher depression and anxiety levels than controls. BMS patients showed lower gray matter volume (GMV) in the bilateral ventromedial prefrontal cortex (VMPFC) and increased functional connectivity between this region and the bilateral amygdala. Region of interest (ROI) analysis suggested that the functional connectivity between the bilateral VMPFC and amygdala correlated with the years of BMS illness in patients. The brain measures could predict the years of symptoms in the BMS group. These results suggest A potential neuromarker for the diagnosis and treatment of BMS.

## Introduction

Burning mouth syndrome (BMS) is a chronic intraoral pain disorder featuring a burning sensation in the tongue or other oral sites in the absence of observable lesions or laboratory findings (Grushka, 1987; Bergdahl and Bergdahl, 1999; Forssell et al., 2002; Grushka et al., 2002; Khan et al., 2014). Patients with BMS have multiple oral complaints, including burning, dryness, and taste alterations (Grushka et al., 2002; Scala et al., 2003). BMS is quite common, with previous studies finding a prevalence of 0.7 and 4.6% in the general population (Bergdahl and Bergdahl, 1999; Khan et al., 2014), and mainly occurs in women after menopause (Bergdahl and Bergdahl, 1999; Grushka et al., 2002). The symptoms of BMS usually start in the morning, increase through the day, and peak in the evening (Grushka, 1987; Bergdahl and Bergdahl, 1999; Grushka et al., 2002).

Due to the lack of lesions in the mouth, BMS has usually been associated with mood problems, such as anxiety or depression (Forssell et al., 2002; Scala et al., 2003). BMS patients usually present with hormonal changes. Some reports have also mentioned that BMS patients have dysfunction of taste sensations (Grushka et al., 2002). However, the etiology of BMS is poorly understood, making both diagnosis and treatment challenging (Khan et al., 2014). For example, although we know that BMS is associated with depression and anxiety, the treatment of mood problems has little impact on BMS *per se* (Grushka et al., 2002; Scala et al., 2003).

Recently, a pioneer study tried to use fMRI as a measure of brain functions for potential use in the diagnosis and treatment of BMS (Khan et al., 2014). The researchers recruited nine female patients and nine matched controls, and they compared the voxel-based morphometry (VBM), diffusion tensor imaging (DTI), and functional connectivity between these two groups. The results suggested that BMS patients had higher gray matter volume (GMV) and lower fractional anisotropy (FA) in the right hippocampus and lower GMV in the left medial prefrontal cortex (mPFC), as well as altered functional connectivity patterns in these regions. Importantly, the subjects were scanned twice, once in the morning when the symptoms were liminal and once in the afternoon when the symptoms were severe. Another study examined the structural changes of BMS with a sample of 12 subjects in each group (Sinding et al., 2016) and found that several pain matrix brain regions showed altered gray matter changes. However, these studies of brain changes in BMS featured a small number of participants, which limited the conclusions that could be drawn from them.

The present study aimed to further elucidate the brain anatomical and functional changes in BMS with a relatively large sample. To simplify the design, the study only scanned participants in the afternoon (between 4 and 5 pm), when there were severe symptoms and a larger change in brain activity (Khan et al., 2014). Trait and state anxiety as well as depression were measured to correlate with the brain activity.

## Materials and Methods

### Participants

The participants were 26 patients with BMS (21 women, mean age = 52.12 ± 8.81 years) and 27 gender- and age-matched control subjects (25 women, mean age = 51.11 ± 5.42 years). They were recruited from the outpatient department in Xiangya Hospital of Central South University in Changsha. The BMS patients met the inclusion criteria: an intraoral burning sensation or dysesthesia; recurring daily for more than 2 h per day over more than 3 months; without clinically evident causative lesions. The control group was recruited from a healthy population. These two groups did not differ in term of age [*t*(51) = 0.50, *p* > 0.05]. The subjects were screened for neurological and psychiatric disorders by a qualified physician using the Mini-Mental State Examination (MMSE). They were also screened for any condition that could disqualify them from fMRI scanning. All research protocols were explained to the participants, and they signed the written consent form approved by the Medical Ethical Committee of the Xiangya Hospital of Central South University of Hunan Province, Changsha, China. Those patients who did not agree to participate in the study were excluded.

### Procedures

Participants came to Xiangya Hospital (Department of Stomatology) to complete the behavior interview and underwent the magnetic resonance imaging (MRI) scan on the same day. All MRI scans were reserved at the same time of day (between 4 and 5 PM). Each scan took about 30 min to complete. During the scan, images for one high-resolution structural scan, one diffusion tensor image, and one session of resting state scan were acquired.

### Behavior Interviews

Patients were diagnosed with BMS before they enrolled in this study. Their history of BMS (in years) was obtained. The Visual Analogue Scale (VAS) was used to measure the severity of pain (Hawker et al., 2011), with 0 = none and 10 = extreme amount of pain. Both patients and controls then completed the State-Trait Anxiety Inventory (STAI; Spielberger, 2010) and the Beck Depression Inventory (BDI; Beck et al., 1996). Their age and gender were then collected during the MMSE interview (Tombaugh and McIntyre, 1992).

### Magnetic Resonance Imaging Protocol

All MRI images were acquired using a General Electric Signa HDx 3.0T scanner at the Xiangya Hospital. Standard settings were used to perform the scan. For example, foam pads were used to minimize head motion. Participants were instructed to relax and keep their head very still during the scan. The structural scan was performed using the T1-FLAIR sequence, covering the whole brain with the following scanning parameters: TR/TE = 1,900/3.0 ms, flip angle = 9°, matrix = 256 × 256, number of slices = 176, slice gap = 0 mm, FOV = 256 mm × 256 mm, slice thickness = 1 mm, bandwidth = 31.25 Hz, sagittal slice position, and the scan lasted 4 min 30 s. DTI scans were performed using a single-shot spin-echo planar image (SE-EPI) sequence with the following parameters: diffusion gradient = 64, number of excitations (NEX) = 2, TR/TE = 12,000/72.4 ms, slice = 50, slice gap = 0, slice thickness = 3.0 mm, FOV = 256 mm × 256 mm, matrix = 128 × 128; the first three volumes were all b0 images, and the rest were images with *b* = 1,000 s/mm^2^, voxel size = 2 mm × 2 mm × 3 mm, and the scan lasted 13 min 30 s. Resting-state functional scans were performed using the GRE-EPI sequence with the following parameters: TR/TE = 2,000 ms/30 ms, flip angle = 80°, FOV = 240 mm × 240 mm, matrix = 64 × 64, number of axial slices = 32, slice thickness = 4 mm, slice gap = 0.6 mm, voxel size = 3.75 mm × 3.75 mm × 3.9 mm, 250 scans were performed with a total scanning time = 8 min 20 s. During the resting state fMRI scan, participants were instructed to keep their eye open and not think something specifically.

### Voxel-Based Morphometry Analysis

Structural MRI data were analyzed with FSL-VBM, an optimized voxel-based morphometry analysis toolbox (Ashburner and Friston, 2000; Good et al., 2001) implemented in FSL (Smith et al., 2004). This method has been proven to be an operator-independent approach because it requires no prior information about the location of possible differences in gray matter. The analysis included the following steps. First, structural images were extracted using BET (Smith, 2002). Next, tissue type segmentation was carried out using FAST4 (Zhang et al., 2001). The resulting gray matter partial volume images were then aligned to the gray matter template in the MNI152 standard space using the affine registration tool FLIRT (Jenkinson and Smith, 2001; Jenkinson et al., 2002), followed by nonlinear registration using FNIRT (Andersson et al., 2007a,b), which used a B-spline representation of the registration warp field (Rueckert et al., 1999; Yuan et al., 2017a). The spatially normalized images were then averaged to create a study-specific template, to which the native gray matter images were registered again using both linear and nonlinear algorithms as described above. The registered partial volume images were then modulated by dividing them with the Jacobian of the warp field to correct for local expansion or contraction. The modulated segmented images, which represent the GMV, were then smoothed with an isotropic Gaussian kernel with a 3 mm standard deviation. Finally, voxel-wise general linear models were used to examine the group differences in gray matter volume. Non-parametric permutation methods (Randomize v2.1 in FSL) were used for inference on statistic maps (Nichols and Holmes, 2002). The null distribution at each voxel was constructed using 10,000 random permutations of the data. Threshold-free cluster enhancement (TFCE) was used to correct for multiple comparisons across the whole brain (*p* < 0.05).

### Tract-Based Spatial Statistics Analysis

The DTI data were processed by FMRIB’s Diffusion Toolbox (FDT) implemented in FSL. Diffusion data were corrected for eddy currents and possible head motion. Images were then skull-stripped (Smith, 2002), aligned to Montreal Neurological Institute (MNI) space using FMRIB’s Nonlinear Registration Tool (FNIRT; Andersson et al., 2007a,b), and resampled to 1 mm^3^. FA was reconstructed by fitting a diffusion tensor model at each voxel. Voxel-wise statistical analysis of the FA data was carried out using the tract-based spatial statistics (TBSS; Smith et al., 2006), part of FMRIB’s Software Library (FSL). The mean FA image was created and thinned to create a mean FA skeleton that represented the centers of all tracts common to the group. Each subject’s aligned FA data were then projected onto this skeleton, and the resulting data were fed into voxel-wise cross-subject statistics. Finally, the group difference between the resulting skeletonized FA images was computed using non-parametric permutation methods (Randomize v2.1 in FSL; Nichols and Holmes, 2002; Yuan et al., 2017b). The null distribution at each voxel was constructed using 10,000 random permutations of the data. TFCE was used to correct for multiple comparisons across the whole brain (*p* < 0.05).

### Functional Connectivity Analysis

The resting-state fMRI data were preprocessed and analyzed using SPM8. The first five volumes were removed for a better fMRI signal. The images were preprocessed in the following steps: slice timing correction; motion correction; co-registration of the anatomical image to the mean functional image; segmentation of the anatomical image CSF, white matter, and gray matter; normalization to standard MNI brain template; and smoothing with an 8 mm Gaussian kernel. Then, seed-based fMRI connectivity was performed using 6 mm-radius spheres around the peak voxels for the VMPFC showing GMV differences between patients and controls. The Conn toolbox (Whitfield-Gabrieli and Nieto-Castanon, 2012) was used to compute the functional connectivity with a bandpass filter of 0.008–0.09 Hz. Six motion-corrected parameters were included in the GLM model. The Pearson correlations were calculated across the whole brain, and group differences were calculated with a two-sample *t* test. False discovery rate (FDR) correction was used for multiple comparisons at voxel level and *p* < 0.05 corrected.

### Correlations With Clinical Variables

To best illustrate the correlations between brain measures and clinical variables (such as depression or history of illness), region of interest (ROI) analysis was performed on significant clusters revealed in the voxel-wise analysis. The Pearson correlations were calculated, and when possible, the scatter plot was illustrated.

## Results

Table 1 shows the demographic characteristics and clinical measures for BMS patients and healthy controls. The results suggested that the two groups were comparable in age and gender; however, the two groups showed significant differences in depression, trait anxiety, and state anxiety. On all three measures, patients showed higher scores than controls, suggesting that BMS patients had significantly higher trait and state anxiety and depression.

**Table 1:** Demographic characteristics and clinical measures of participants (*M* ± SD).

	BMS patients	Healthy controls	Statistics
*n* (females)	26 (21)	27 (25)	*χ*^2^(1) = 1.62, *p* = 0.20
Age (years)	52.12 ± 8.81	51.11 ± 5.42	*t*(51) = 0.50, *p* = 0.62
Depression (BDI)	11.27 ± 6.50	2.59 ± 2.06	*t*(51) = 6.60, *p* < 0.001
Trait anxiety (STAI)	38.23 ± 14.30	24.30 ± 4.59	*t*(51) = 4.82, *p* < 0.001
State anxiety (STAI)	36.12 ± 13.35	24.11 ± 4.71	*t*(51) = 4.40, *p* < 0.001
BMS history (months)	8.61 ± 9.93	–	–
VAS pain rating	4.19 ± 1.60	–	–

### Voxel-Based Morphometry Results

**Figure 1:**
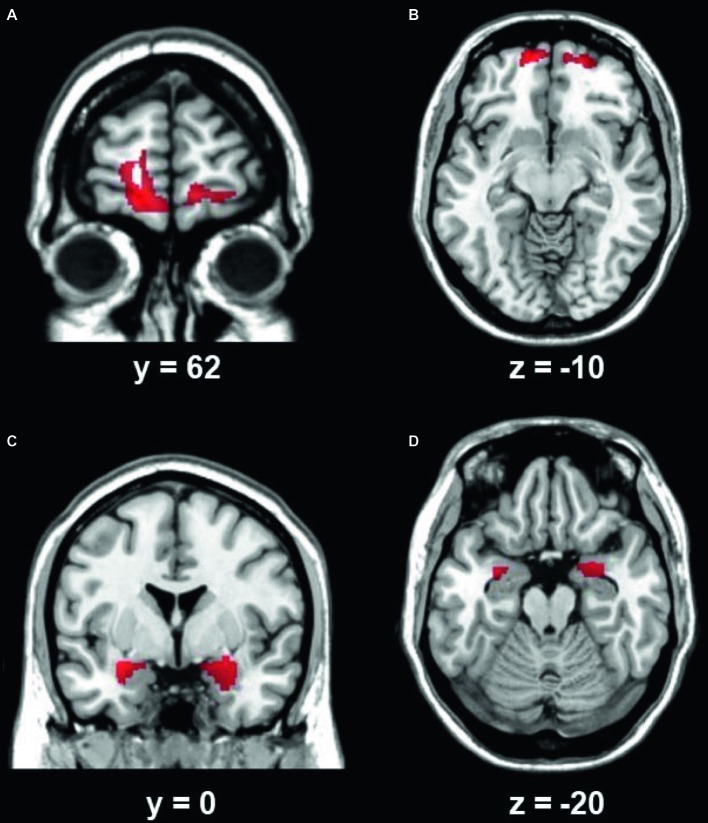
The left **(A)** and right **(B)** VMPFC showed lower gray matter volume in BMS patients than in controls. The left **(C)** and right **(D)** amygdala showed increased functional connectivity in BMS patients than in controls. The results were mapped onto the standard brain for visual display, with the left side representing the right hemisphere.

VBM results suggested that there were two clusters (Table 2; Figures 1A,B) showing significantly lower gray matter volume in patients than in controls, including the left and right ventromedial prefrontal cortex (VMPFC). No region showed significantly higher GMV in patients than in controls. ROI analysis suggested that the GMV in the bilateral VMPFC correlated with the years of BMS illness in patients [left: *r*(26) = −0.81, *p* < 0.001; right: *r*(26) = −0.80, *p* < 0.001].

**Table 2:** Summary of MRI results.

Brain region		No. of voxels	Peak voxel			Geometry center			*Z*
			MNI *x*	MNI *y*	MNI *z*	MNI *x*	MNI *y*	MNI *z*	
**VBM results (patients < controls)**									
L	VMPFC	196	−12	60	−8	−11.9	60.9	−7.1	5.76
R	VMPFC	115	22	58	−10	19.8	59	−8.73	4.54
**Functional connectivity results (patients > controls)**									
R	Amygdala	190	28	2	−14	25	−0.74	−18.5	5.27
L	Amygdala	79	−28	0	−14	−27.5	−1.77	−18.1	5.62

*L, left; R, right; MNI, Montreal Neurological Institute; VMPFC, ventromedial prefrontal cortex*.

### Tract-Based Spatial Statistics Results

TBSS did not reveal any significant difference between patients and controls with regard to FA and MD.

### Functional Connectivity Results

Using the left and right VMPFC as the seed region, whole-brain connectivity analysis suggested that the bilateral amygdala had increased functional connectivity with the bilateral VMPFC region (Table 2; Figures 1C,D) in patients than in controls. There was no decreased functional connectivity in patients compared to controls. ROI analysis suggested that the functional connectivity between the bilateral VMPFC and amygdala correlated with the years of BMS illness in patients [left: *r*(26) = 0.71, *p* < 0.001; right: *r*(26) = 0.68, *p* < 0.001].

## Discussion

For the present study, we recruited 26 BMS patients and 27 healthy controls to investigate the possible deficits of brain structure and function in BMS patients. The results revealed that BMS patients had higher levels of depression and anxiety than controls. BMS patients showed lower GMV in the bilateral VMPFC and increased functional connectivity between this region and the bilateral amygdala. Region of interest (ROI) analysis suggested that the functional connectivity between the bilateral VMPFC and amygdala correlated with the years of BMS illness in patients. The brain measures could predict the years of symptoms in the BMS group.

These results replicated those of previous studies on the mood problems in BMS patients (Grushka, 1987; Bergdahl and Bergdahl, 1999; Forssell et al., 2002; Grushka et al., 2002; Scala et al., 2003; Khan et al., 2014; Sinding et al., 2016). In our study, depression, trait anxiety, and state anxiety were all higher in BMS patients than in controls. Although the scores of the BMS group were comparable to those found in previous studies (Khan et al., 2014), we found even lower anxiety scores in the controls. This may be because our sample was from a nearby community, while previous studies only recruited controls from other departments of the hospital.

Additionally, our results suggested that BMS patients had lower GMV in the bilateral VMPFC region, and the GMV of this region was inversely correlated with the severity of BMS. These results partially replicated the results of Khan et al. (2014) but were not consistent with a later study by Sinding et al. (2016). The inconsistency may be partially due to the smaller sample size used in the previous studies. Decreased GMV in the VMPFC has been reported previously in other chronic pain populations (Ploghaus et al., 2003; Kong et al., 2010; Yu et al., 2014). Besides chronical and social pain (Ploghaus et al., 2003; Masten et al., 2011), this region is known to be involved in many other functions, such as decision making (Koritzky et al., 2013; Zhu et al., 2018; He et al., 2019; Huang et al., 2019), stress regulation and inhibition (Dickie et al., 2011; Conner et al., 2012; Shvil et al., 2014), and emotion processing (Northoff et al., 2004; Grimm et al., 2006; Moll and de Oliveira-Souza, 2007). The decreased GMV in the bilateral VMPFC may be a neuromarker for the diagnosis and treatment of BMS.

Our results further indicated that the functional connectivity between the VMPFC and amygdala was increased in BMS patients, and this phenomenon was associated with the severity of BMS. This is consistent with previous studies (Hawker et al., 2011) showing functional connectivity enhancement between the mPFC and amygdala for more severe conditions (comparable to our afternoon session). The amygdala has long been linked to emotional processing (Morris et al., 1998; Davis and Whalen, 2001; Hamann and Mao, 2002; LeDoux, 2003), especially fear (LeDoux, 2003). The amygdala has also been linked to substance addiction and behavior addiction, such as social media addiction (He et al., 2017; Zhu et al., 2017a). Previous reports have suggested that the functional connectivity between the amygdala and medial PFC is associated with anxiety (Kim et al., 2011), emotion regulation (Banks et al., 2007; Zhu et al., 2017b), emotional learning (Sakaki et al., 2016), and sleep (Yoo et al., 2007). In summary, the increased functional connectivity between the amygdala and VMPFC may be another neuromarker for the diagnosis and treatment of BMS.

Three limitations of the current study should be noted. First, the study used a relatively large sample but without a balance of males and females. Although BMS is a female-dominated disease, future studies should include more male participants to generalize the findings to the whole population. Second, the cross-sectional methods used in this study limit our inference of the causality. Future studies with different data samples collected during the day or longitudinal studies would be very useful because of the dynamic changes in BMS symptoms. Meanwhile, further longitudinal studies should be conducted to replicate and expand on the findings of this study. Third, this study only investigated the stable brain signals (e.g., the GMV); future studies should use fMRI designs with different types of stimulation to investigate the dynamic patterns of brain activity.

## Data Availability

All datasets generated for this study are included in the manuscript and/or the supplementary files.

## Ethics Statement

All participants were aware of the purpose of the study and signed an informed consent before the study. All research protocols were explained to the participants, and they signed the written consent form approved by the Medical Ethical Committee of the Xiangya Hospital of Central South University of Hunan Province, Changsha, China. Informed consent was obtained from all individual participants included in the study.

## Author Contributions

YT and LK conceived and designed the experiments. YT, XW, and JC conducted the experiments and collected the data. LK and ZQ analyzed the results. LK and ZQ wrote the main manuscript text. All authors reviewed the manuscript.

### Conflict of Interest Statement

The authors declare that the research was conducted in the absence of any commercial or financial relationships that could be construed as a potential conflict of interest.
